# A Rare Case of Concurrent Intramedullary and Brain Tuberculoma: Diagnostic Challenges and Management

**DOI:** 10.7759/cureus.75537

**Published:** 2024-12-11

**Authors:** Jeevitha Bhanoth, Aruba Usman, Haindavi Bandari, Syeda Areeba Junaid, Noorin Sultana

**Affiliations:** 1 Department of General Medicine, Osmania General Hospital, Hyderabad, IND; 2 Department of Internal Medicine, Osmania Medical College, Hyderabad, IND

**Keywords:** anti-tubercular therapy, intramedullary tuberculoma, quadriparesis, retroviral disease, seizures, target sign

## Abstract

Intramedullary spinal tuberculomas constitute a small percentage of spinal tuberculosis. These, in combination with brain tuberculomas, are an uncommon manifestation of central nervous system (CNS) tuberculosis. This report details a unique case of a 32-year-old retroviral disease-positive male who presented with a two-month history of symmetrical quadriparesis and recent seizures. The neurological assessment revealed significant weakness, with a power of 3/5, associated with stiffness, hypoesthesia below the neck, and urinary hesitancy. He was diagnosed via magnetic resonance imaging (MRI), which showed a characteristic target sign at the C2 level and a hyperintense lesion in the left high parietal lobe. In a multi-faceted approach, the patient received a 12-month regimen of five-drug anti-tubercular therapy, supplemented with physiotherapy and continuous antiretroviral treatment. This case highlights the crucial need to consider tuberculoma as a differential diagnosis in patients presenting with similar symptoms, while also keeping conditions such as toxoplasmosis, CNS lymphoma, and glioblastoma in mind. Early detection and intervention are vital, as prompt treatment can significantly change the trajectory of this debilitating disease, potentially saving lives and restoring neurological function.

## Introduction

Tuberculomas, also known as tuberculous granulomas, are well-defined focal lesions caused by *Mycobacterium tuberculosis*. They are one of the more severe morphological manifestations of tuberculosis [1]. Pathologically, tuberculomas are space-occupying granulomas that develop as a result of the body's inflammatory response to the tuberculous bacilli [2]. Although intramedullary tuberculomas are uncommon, spine involvement in tuberculosis is widespread [3].

Global tuberculosis incidence in 2022 is predicted to be 10.6 million cases, according to the WHO. The National Tuberculosis Elimination Programme’s annual report on tuberculosis in India states that there were 24.2 lakh cases registered in 2022 - a 13% rise over 2021. In India, the prevalence of spinal tuberculosis is 4.8%. Individuals who are human immunodeficiency virus (HIV)-positive have an 18-fold increased risk of developing tuberculosis, with an incidence of 54,000 cases in India and 703,000 cases globally [4]. According to estimates, half of patients with disseminated tuberculosis and about one out of every 300 untreated pulmonary tuberculosis cases acquire tuberculosis in the brain parenchyma. About 25% to 30% of patients with brain tuberculosis are not impacted by pulmonary tuberculosis, despite several studies reporting that almost 75% of patients developed pulmonary tuberculosis 6 to 12 months before being diagnosed with central nervous system (CNS) tuberculosis [5]. Only around 10% of people with systemic tuberculosis have involvement of the CNS [6]. Only 0.2% to 5% of all CNS tuberculomas are intramedullary, making them extremely uncommon [7]. In cases of spinal tuberculosis, the majority of patients (55%) present with vertebral body involvement, while 39% exhibit intraspinal granulomatous lesions without bone involvement. Only a small percentage - 7% - show intramedullary lesions [8]. The occurrence of both intramedullary and intracranial tuberculomas together is exceptionally uncommon, with only a few cases documented in the existing literature [7].

We report a case of a 32-year-old retroviral disease-positive male who presented with insidious onset and gradually progressive quadriparesis. He was diagnosed with intramedullary tuberculoma at the C2 level, along with a parietal lobe lesion. Treatment includes medical management with antituberculosis therapy for 12 to 18 months.

## Case presentation

A 32-year-old retroviral disease-positive male patient presented with symmetrical weakness in both the upper and lower limbs for the past two months. The weakness was progressive, beginning in the right upper limb and lower limb, followed by the involvement of the left upper limb and lower limb. It was associated with stiffness. The patient experienced episodes of tripping. The weakness was more pronounced in the distal muscles of the limbs than in the proximal muscles. There was no history of root pain, band-like sensations, or local pain. There was a history of hypoesthesia below the level of the neck. The patient also had complaints of hesitancy and incomplete voiding of urine. There was no history of recent head trauma or recent vaccination. He also had a history of seizures, with two to three episodes per day over the past week, involving tonic flexion of the right upper limb and lower limb. The seizures lasted for one minute and were not associated with preceding aura, premonitory symptoms, or impaired awareness. There was no post-ictal confusion, tongue bite, or urinary incontinence. There was no history of cranial nerve deficits. There was no complaint of recent upper respiratory tract infections or gastroenteritis. There was no history of headache, fever, vomiting, drug abuse, altered sensorium, or blurring of vision. There was no history of similar complaints in the past. The patient was a known case of retroviral disease for the past three years, on antiretroviral therapy for one year, and non-compliant with medication. The patient also had a history of pulmonary tuberculosis and used antitubercular therapy for six months, with no resistance to rifampicin or isoniazid. However, the patient was non-compliant with the medication, and the course was completed three months ago.

The patient was thin-built, and the general examination was normal. The cardiovascular system, gastrointestinal system, and respiratory system examinations were normal. On neurological examination, the patient was conscious and coherent, with a Mini-Mental State Examination (MMSE) score of 25, and no cranial nerve difficulties. Motor examination revealed hypertonia in all four limbs and a Medical Research Council (MRC) grade of 3/5 power in all four limbs. The deep tendon reflexes were exaggerated in all four limbs, with bilateral ankle clonus and a bilateral extensor plantar response. No cerebellar abnormalities were noted. Sensory abnormalities, including decreased sensation of pain, touch, and temperature below the neck (C2), were noted. There were no spinal deformities or spinal tenderness. No local rise in temperature, no scars, no sinuses, and no signs of meningeal irritation were present.

The complete blood picture (CBP) showed neutrophilic leukocytosis; liver function tests and renal function tests were normal. HIV-1 antigen was reactive, with a CD4 count of 150 cells/mm^3^. Hepatitis B surface antigen (HBsAg), hepatitis C virus (HCV), and venereal disease research laboratory (VDRL) were nonreactive. The erythrocyte sedimentation rate (ESR) was elevated to 100 mm/hr. The Mantoux test showed no induration. Cartridge-based nucleic acid amplification test (CBNAAT) (GeneXpert; Cepheid, Sunnyvale, CA, USA) from cerebrospinal fluid (CSF) detected *M. tuberculosis* and showed sensitivity to rifampicin. CSF analysis, as shown in Table 1, revealed pleocytosis with 240 cells (80% lymphocytes), mildly raised proteins at 50 mg/dL, and low sugar levels at 30 mg/dL. The India ink stain of CSF for *Cryptococcus* was negative.

**Table 1:** CSF analysis values CSF, cerebrospinal fluid

CSF analysis	Observed values	Normal values
Volume	10 mL	90 to 150 mL
Color	Transparent	Clear, colorless
Turbidity	Not seen	Absent
Coagulum	Not found	Absent
Glucose	30 mg/dL	50 to 80 mg/dL
Total proteins	50 mg/dL	15 to 45 mg/dL
Cell count	150 cells/mm^3^	Less than 5 cells
Neutrophils	30 cells/mm^3^	Not seen
Lymphocytes	120 cells/mm^3^	Not seen

**Figure 1:**
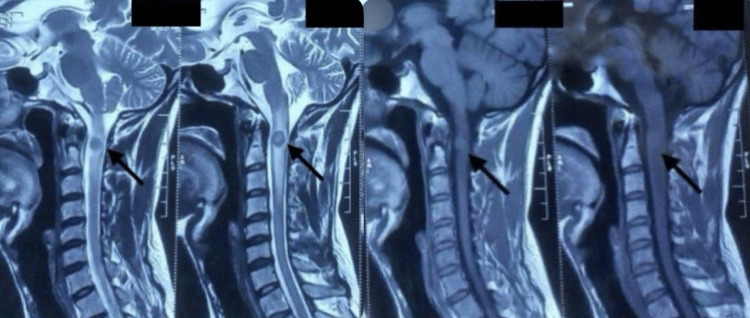
T2 weighted image (left two images), T1 weighted image (right two images). The left two images show T2 central hyperintensity, and the surrounding T2 hypointense rim giving a target pattern as indicated by the arrows. The right two images show a well-defined, oval, tiny lesion, which was hypointense on T1 as indicated by the arrows.

**Figure 2:**
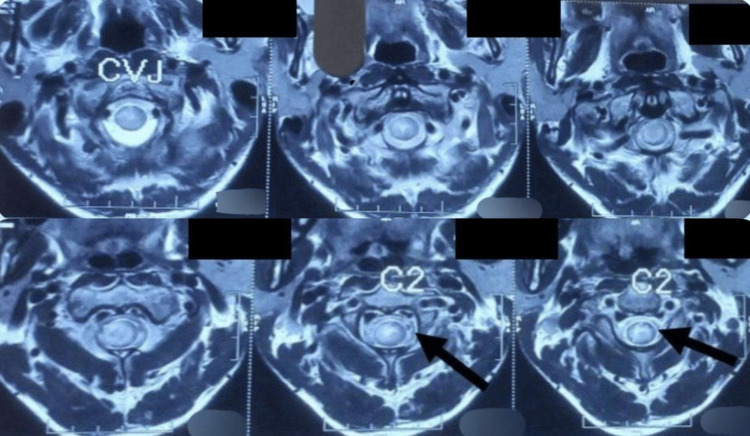
T2 axial section of the spine showing target pattern. Target pattern noted eccentrically in intramedullary space of spinal cord at C2 level as indicated by the arrows in the T2 axial section.

**Figure 3:**
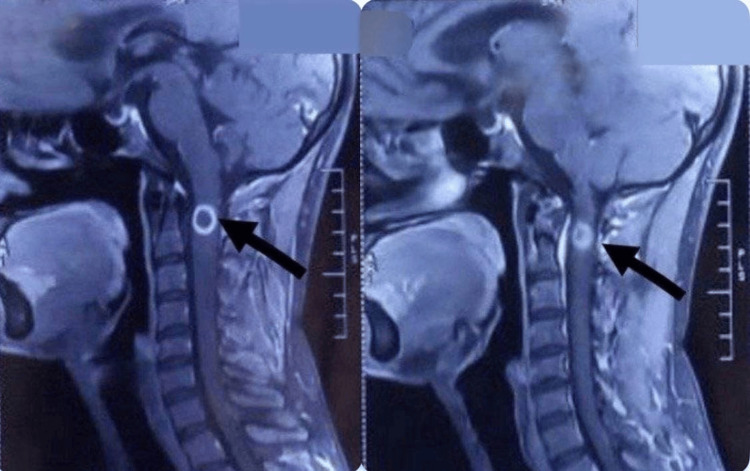
Post-contrast image of the spine. Post-contrast image showing smooth ring enhancement as indicated by the arrows.

**Figure 4:**
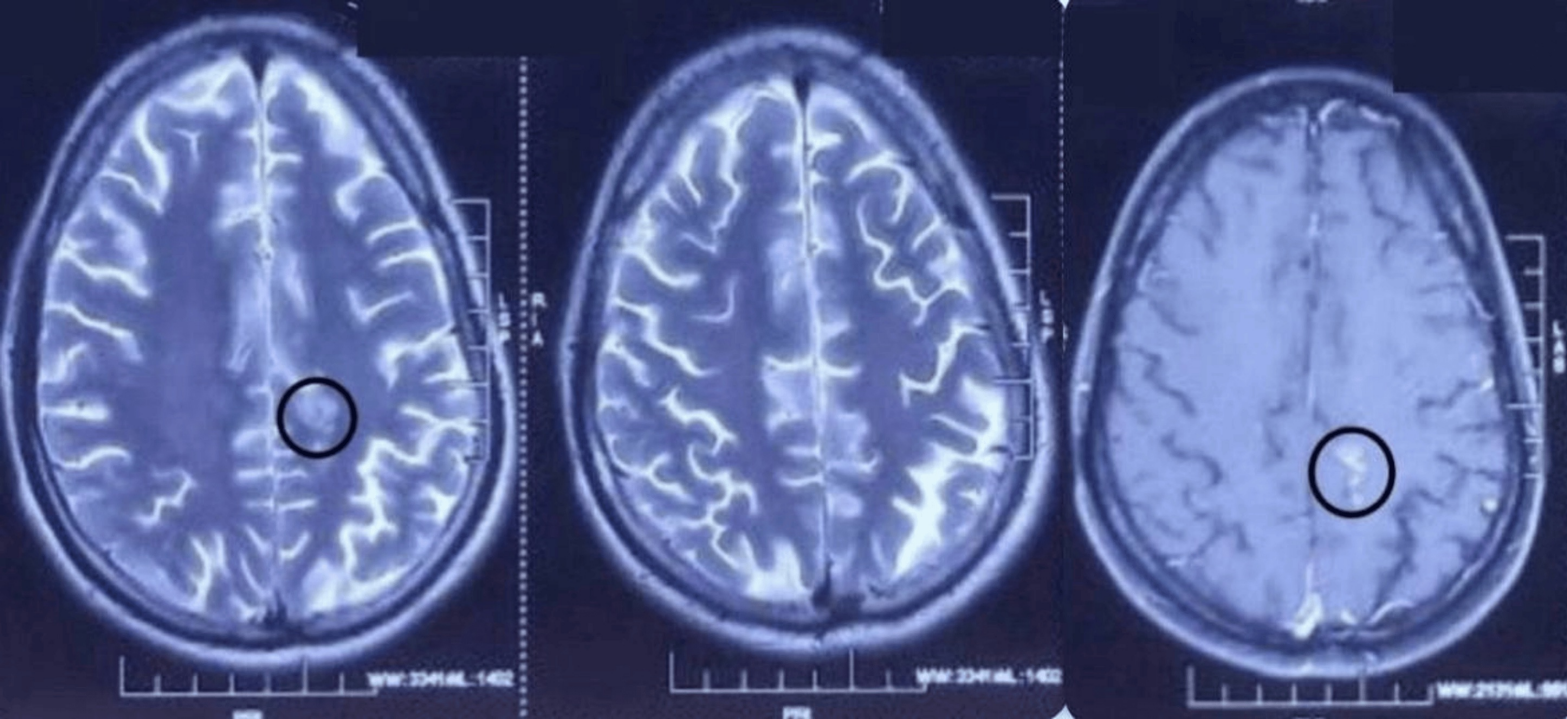
T2 axial section of the brain. T2 axial section of the brain showing small T2 hyperintense lesion with corresponding hypo intensity in the left high parietal lobe as indicated by the circle.

The magnetic resonance imaging (MRI) of the spine showed evidence of a well-defined, oval, tiny lesion, which was hypointense on T1 and T2, with central hyperintensity and a surrounding T2 hypointense rim, giving a target pattern, as shown in Figure 1. This lesion was noted eccentrically in the intramedullary space of the spinal cord at the C2 level, as shown in Figure 2. Post-contrast imaging revealed smooth ring enhancement of the lesion, as shown in Figure 3. Evidence of T2/short tau inversion recovery (STIR) hyperintense cord edema was noted adjacent to the lesion, extending from the corticomedullary junction to the C4 level. The MRI of the brain showed evidence of a small T2 hyperintense lesion with corresponding hypointensity noted in the left high parietal lobe, showing homogeneous post-contrast enhancement, without diffusion restriction or adjacent edema, as shown in Figure 4. A radiological diagnosis of spinal intramedullary tuberculoma at the C2 level, along with a left high parietal lobe tuberculoma, was made.

The patient was started on a five-drug anti-tubercular therapy for 12 months, including isoniazid (300 mg/day), rifampicin (450 mg/day), ethambutol (800 mg/day), pyrazinamide (1500 mg/day), and injection streptomycin (0.75 g/day), along with pyridoxine (40 mg/day) for two months. This was followed by isoniazid (300 mg/day), rifampicin (450 mg/day), and pyridoxine (40 mg/day) for 10 months. Injectable steroid dexamethasone in a tapering dose was given for two months, and carbamazepine 300 mg was given twice daily. The patient was advised physiotherapy and to continue the anti-retroviral therapy regimen.

At discharge, the patient had a power of 4/5 in all limbs, with no seizure episodes. The patient was advised to revisit for follow-up after two months of treatment. He was counseled on strict compliance with medication.

## Discussion

Hematogenous dissemination of *M. tuberculosis* from an extracranial source is the cause of tuberculosis of the CNS [5]. As reported by Kumar et al., intraspinal tuberculoma accounts for around 1% of cases of spinal tuberculosis [9]. Different subtypes of intraspinal tuberculoma exist based on the location of the lesion, such as intramedullary, intradural, extramedullary, and epidural tuberculomas [10]. The majority of intramedullary tuberculosis cases have subacute symptoms, which worsen over time and point to compressive myelopathy. Intramedullary tuberculoma can cause muscle weakness, sensory loss, or loss of autonomic function, depending on which spinal level is damaged [11].

Radiological investigations suggest significant inflammation that results in edema, which is a hallmark of the early stages of tuberculoma, as the gel capsule is not perfectly formed at this point. At this stage, the enhancement following the contrast examination is consistent. The signal intensity on the T1-weighted image (T1WI) and T2-weighted image (T2WI) is the same. Peripheral edema starts to go away as the amount of gel in the tuberculoma grows. Consequently, low or isointense lesions are shown on T2WI, whereas isointense lesions are shown on T1WI. The contrast-enhanced MRI displays central hypointensity. T2WI displays a typical "target sign" with caseation development, meaning that it measures from the center of the low-signal rim to the peripheral areas, as well as from the low-signal target to the high-signal rim. The target sign characteristic is given by the caseous substance appearing hyperintense at the center. Collagen fibers made by fibroblasts make up the low-signal rim in the outer region. When distinguishing spinal tuberculoma from other intramedullary lesions, the target sign is a relevant indicator [12]. Symptoms similar to those in our case, along with comparable MRI findings, such as rim-enhancing lesions, lead us to consider differential diagnoses including toxoplasmosis, brain abscess, CNS lymphoma, and glioblastoma. Toxoplasmosis often presents on MRI with variable T2 signal intensity, ranging from hyperintense to isointense. Characteristically, it displays a concentric target sign with alternating zones of hypo/hyper/isointense signals, accompanied by perilesional edema. On T1WIs with gadolinium contrast, lesions typically show ring or nodular enhancement, often featuring an eccentric nodule. In contrast, abscesses typically exhibit T1 hypointensity and T2 central hyperintensity, surrounded by a hypointense rim. T1WIs with contrast reveal peripheral enhancement, while diffusion-weighted imaging (DWI) shows high signal intensity, with apparent diffusion coefficient (ADC) values being lower compared to the surrounding spinal cord. CNS lymphoma has distinct MRI characteristics: T1WIs are generally hypointense, while T2WIs are characteristically hypointense in the brain but hyperintense in the spinal cord. In immunocompetent patients, contrast-enhanced imaging shows homogeneous solid enhancement, whereas immunocompromised patients often display heterogeneous ring enhancement. Lastly, glioblastoma is identified by T1 hypointensity and T2 hyperintensity, with notable heterogeneity in signal and irregular lesion outlines. An irregular ring enhancement, frequently accompanied by a thick enhancing rim, is a hallmark feature of glioblastoma. Considering these differential diagnoses is crucial for accurate identification and effective management of CNS lesions.

Although radiological imaging facilitates diagnosis, a biopsy is thought to be the gold standard for diagnosis. Biopsy is used in cases of drug-resistant tuberculosis, when non-invasive testing has not been able to aid in diagnosis, when patients fail to respond to a treatment regimen, or in patients who do not comply with the treatment [5]. The commonly seen biopsy findings in a similar case report included: a necrotizing granulomatous inflammation encircled by pale histiocytes. According to the histological and imaging results, the lesion was suspected to be spinal tuberculosis. Ziehl-Nelsen and Grocott methenamine silver stains, among other special stains used, were negative. The biopsy specimen was subjected to additional staining, such as Fite stain for acid-fast bacilli and periodic acid-Schiff for fungi. The results of these stains confirmed the suspected diagnosis of intramedullary spinal tuberculoma, as rare acid-fast bacilli were identified on the Fite stain [13]. These findings can help predict the likely course of the illness and guide a treatment plan. They can also provide possible outcomes and management approaches to the patient and the healthcare team.

Some authors have observed that 78% of brain tuberculomas resolved within 12 to 24 months of treatment, whereas 22% required more than 24 months. However, a median resolution time of 36 months is observed in cases involving multiple tuberculomas. In cases where a brain tuberculoma exceeds 20 mm in size or causes a mass effect on the brain, surgery might be necessary. Similar to other lesions occupying space, an elevated intracranial pressure signals a surgical emergency, as does the complete failure of medical therapy [5]. An early diagnosis and course of treatment are particularly beneficial for spinal tuberculosis. Regarding the treatment of spinal tuberculosis, there is no conclusive recommendation. If the lesion is accessible, or if there is spinal instability, the common agreement is aggressive medical management followed by surgery [14]. The primary form of treatment for intramedullary tuberculoma involves anti-tubercular therapy [15]. The typical treatment for tuberculosis involves two main phases: an initial stage with multiple medications to quickly reduce the bacterial load and address drug-resistant strains, followed by a second phase with fewer drugs to eliminate any remaining "dormant" bacteria and prevent a relapse of the illness [13]. The WHO recommends a two-phase treatment for drug-susceptible CNS tuberculosis. The intensive phase lasts for two months and is followed by a continuation phase of 7-10 months. During the intensive phase, the patient is given four first-line medications: isoniazid, rifampicin, pyrazinamide, and streptomycin. In the continuation phase, the patient receives a two-drug regimen of isoniazid and rifampicin. The British Infection Society and the National Institute for Health and Care Excellence (NICE) guidelines advise at least 12 months of treatment for all types of CNS tuberculosis. This includes isoniazid and rifampicin for the first two months, followed by pyrazinamide, ethambutol, and isoniazid for the remaining 10 months. According to the NICE guidelines, spinal cord involvement from tuberculosis should be treated as CNS tuberculosis. The American Thoracic Society suggests 9-12 months of treatment for meningeal involvement, including isoniazid, rifampicin, pyrazinamide, and ethambutol for the first two months, followed by isoniazid and rifampicin for 7-10 months. Similarly, the Indian Index-Tuberculosis guidelines recommend treating CNS tuberculosis with isoniazid, rifampicin, pyrazinamide, and ethambutol for two months, then isoniazid, rifampicin, and ethambutol for seven months. There is uncertainty about the benefit of continued anti-tubercular therapy for patients with severe spinal arachnoiditis [16]. Before the introduction of MRIs, the primary approach for diagnosing and treating intramedullary abscess and tuberculoma involved surgically removing the intramedullary lesion. Presently, the most effective treatment for intramedullary tuberculoma and abscess involves anti-tubercular therapy. Additionally, the use of supplementary corticosteroid medication has beneficial outcomes. Early detection and treatment can help avoid complications and unnecessary surgical procedures [16]. Surgical intervention should only be considered in specific circumstances, such as severe neurological deficits, inadequate response to treatment, deterioration in neurological condition despite medical attention, or an unexpected enlargement of the lesion observed on a follow-up MRI [17]. According to Gupta et al.'s extensive experience of over 30 years, they concluded that surgical intervention was not necessary, even for patients in advanced stages of the disease, such as retropharyngeal abscess with or without atlantoaxial dislocation, severe bone destruction, or angulation [18]. They also mentioned that all 51 patients with cranio-vertebral junction tuberculosis, regardless of clinical radiologic grade, could undergo conservative treatment with extended anti-tubercular therapy (18 months) and external rigid immobilization. They observed that even in advanced cases, bone regrowth and alignment restoration occurred after 18 months of anti-tubercular therapy [19]. Our patient was prescribed a five-drug anti-tubercular therapy for 12 months, along with an injectable steroid in tapering doses for two months, and carbamazepine (300 mg twice daily) was administered. He was advised to continue his anti-retroviral therapy regimen.

Intramedullary tuberculoma is an exceptionally rare form of tuberculosis. The diagnosis of intramedullary tuberculoma can be challenging due to its nonspecific symptoms and could be mistaken for other spinal cord lesions. Documenting rare cases can stimulate further research into the pathophysiology, diagnosis, and treatment of intramedullary tuberculoma, which could lead to improved patient care and outcomes in the future.

## Conclusions

This case report highlights the occurrence of an intramedullary tuberculoma at the C2 level of the spinal cord, accompanied by involvement of the left parietal lobe. This condition can present a diagnostic challenge due to its similarity to neoplastic and other inflammatory lesions. Early recognition through patient history, investigations, and radiological imaging is crucial for initiating appropriate anti-tubercular therapy, which can lead to favorable outcomes. The patient in our case, at discharge, showed clinical improvement through medical management using anti-tubercular therapy drugs, consistent with the outcomes reported in similar cases with intramedullary tuberculoma. With careful monitoring, ongoing adherence to the prescribed regimen, and regular imaging follow-ups, we expect continued improvement in our patient. This case underscores the importance of considering tuberculoma in the differential diagnosis of intramedullary spinal cord lesions, particularly in regions endemic to tuberculosis or in immunocompromised patients.
